# PPAR Gamma and Viral Infections of the Brain

**DOI:** 10.3390/ijms22168876

**Published:** 2021-08-18

**Authors:** Pierre Layrolle, Pierre Payoux, Stéphane Chavanas

**Affiliations:** Toulouse NeuroImaging Center (ToNIC), INSERM/UPS UMR 1214, CHU Toulouse-Purpan, 31024 Toulouse, France; pierre.layrolle@inserm.fr (P.L.); pierre.payoux@inserm.fr (P.P.)

**Keywords:** PPAR gamma, brain, neural stem cells, infection, neuroinflammation, HIV, Zika, cytomegalovirus, neurogenesis, microglia

## Abstract

Peroxisome Proliferator-Activated Receptor gamma (PPARγ) is a master regulator of metabolism, adipogenesis, inflammation and cell cycle, and it has been extensively studied in the brain in relation to inflammation or neurodegeneration. Little is known however about its role in viral infections of the brain parenchyma, although they represent the most frequent cause of encephalitis and are a major threat for the developing brain. Specific to viral infections is the ability to subvert signaling pathways of the host cell to ensure virus replication and spreading, as deleterious as the consequences may be for the host. In this respect, the pleiotropic role of PPARγ makes it a critical target of infection. This review aims to provide an update on the role of PPARγ in viral infections of the brain. Recent studies have highlighted the involvement of PPARγ in brain or neural cells infected by immunodeficiency virus 1, Zika virus, or human cytomegalovirus. They have provided a better understanding on PPARγ functions in the infected brain, and revealed that it can be a double-edged sword with respect to inflammation, viral replication, or neuronogenesis. They unraveled new roles of PPARγ in health and disease and could possibly help designing new therapeutic strategies.

## 1. Introduction

Peroxisome Proliferator-Activated Receptor gamma (PPARγ) was discovered and cloned almost 30 years ago, as a new member of a family of receptors activated in response to treatment of liver cells by an heterogeneous group of chemicals, namely peroxysome proliferators [1]. Since then, an ever growing body of research has provided us with a better knowledge about PPARγ, which is now known as a master regulator of gene expression in lipid and glucose metabolism, adipogenesis, inflammation, cell proliferation and cancer [2].

It has been almost 25 years since PPARγ transcripts were detected in brain of rat embryos [3]. This early finding suggested that PPARγ might be of importance in brain development; an assumption that was strengthened thereafter by the observation of a «disorganized brain» in *Pparg* knock-out mouse embryos [4]. PPARγ in the brain has been extensively studied in relation to inflammation or neurodegeneration [5]. A wealth of in vitro, in vivo and clinical studies have shown that PPARγ plays a beneficial role on brain injury [6] and neurodegenerative disorders such as Multiple Sclerosis, Alzheimer’s disease and Amyotrophic Lateral Sclerosis [7]. Also, on the bases of encouraging preclinical studies, PPARγ has been proposed as a possible therapeutic target for psychiatric disorders [8] or drug addiction and substance abuse [9]. Although the role of PPARγ in the regulation of the immune response and inflammation is well established, little is known however about its role in infections of the brain parenchyma, particularly viral infections.

A wide range of different neurotropic viruses cause infections of the adult or developing brain and underlie acute or chronic neuropathies worldwide [10]. Viral infections of the brain represent the most frequent cause of encephalitis, a neurological disorder characterized by acute fever, seizures, neurologic deficits and/or altered behaviour, which affects 7 people out of 100,000 in the U.S.A. each year [11]. Viral congenital infections may have devastating outcomes on the structure and function of the developing brain, or may result in mild to severe lifelong unabilities [12].

A better understanding on the role of PPARγ in the infected brain may help designing new therapeutic strategies. Furthermore, specific to viral infections is the ability to subvert signaling pathways of the host cell in order to ensure viral replication and spread, as deleterious as the consequences may be for the host. For example, many viruses have evolved mechanisms to regulate positively or negatively activity of the nuclear factor ĸB (NF-ĸB) to facilitate their replication, host cell survival, or immuno-evasion [13]. In this respect, the pleiotropic role of PPARγ makes it an expected critical target of infection. Thus, investigating PPARγ in neural cell infections can provide insight on the molecular and cellular outcomes of PPARγ activity in the healthy cell as well as the infected cell.

This review aims to provide for the first time an update on our knowledge of the role of PPARγ in viral infections of the brain parenchyma. It will update the current knowledge on PPARγ molecular aspects and brain expression, point out recent advances about PPARγ focusing on specific brain issues, and, finally, summarise and discuss knowledge on PPARγ and viral infections of the brain parenchyma.

## 2. PPARγ Molecular Levers

Peroxysome proliferator-activated receptors (PPARs) are members of the nuclear receptor superfamily [14]. As such, they are activated by lipophilic, membrane-permeant, ligands. Upon ligand binding, nuclear receptors form homo- or hetero-dimers and translocate to the nucleus to regulate gene transcription. PPAR family comprises three members, PPARα, PPARβ/δ, and PPARγ. They share a common structure containing six highly conserved functional domains: a first transcription activation function domain (AF-1), a two zinc-fingers DNA binding domain (DBD), a hinge domain, a ligand binding domain (LBD) and a second activation function domain (AF-2) that modulates binding to either co-activator or repressor factors in a ligand-dependent fashion [14,15]. The gene encoding PPARγ, namely *PPARG*, has a complex pattern of expression. Two alternative promoters and alternative splicing events can generate seven *PPARG* transcripts translated to two PPARγ isoforms: the widely expressed PPARγ1 and the adipocyte-restricted PPARγ2 [2].

**Figure 1:**
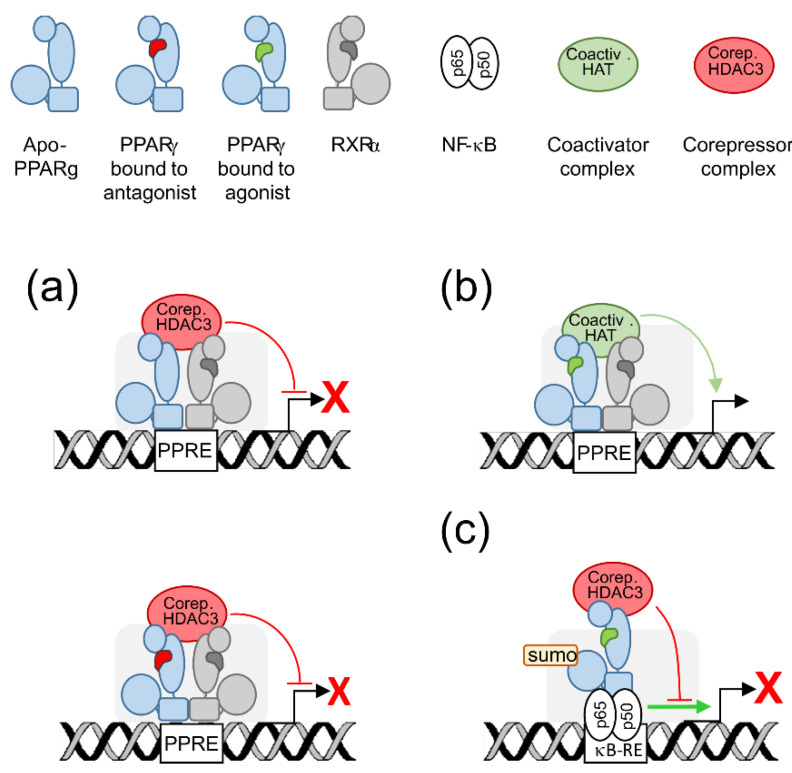
Graphical summary of PPARγ transactivating and transrepressing activities. (**a**) DNA binding-dependent transrepression: unbound PPARγ (top) or PPARγ bound to an antagonist (bottom) forms a dimer with RXRα, binds to a cognate response element (PPRE) and recruits corepressors (Corep.) and HDAC3 to assemble a repressive complex which blocks transcription of the cognate gene (broken arrow). (**b**) Transactivation: agonist-bound PPARγ and RXRα, bound to a PPRE, recruit coactivators (Coactiv.) and HAT to assemble a permissive complex which enhances transcription of the cognate gene. (**c**) DNA binding-independent transrepression: ligand-activated PPARγ and corepressor complex bind to a target transcription factor as NF-ĸB to prevent it from activating cognate gene transcription. Sumoylation (sumo) increases the stability of the complex PPARγ-corepressor. ĸB-RE: NF-ĸB response element.

Transactivation and transrepression refer to positive or negative gene transcriptional regulation by PPARγ, respectively. Transactivation requires both DNA-binding and agonist-binding whereas transrepression may require or not DNA-binding (Figure 1).

PPARγ forms dimers with another nuclear receptor, namely the retinoid X receptor alpha (RXRα) whose ligand is 9-cis retinoic acid [16]. The PPARγ-RXRα dimer translocates to the nucleus and binds cognate DNA sequences named PPAR Responsive Elements (PPRE) [17]. For transactivation, the dimer formed by agonist-bound PPARγ and RXRα recruits coactivators such as PPARγ coactivator 1-α (PGC-1α), E1A binding protein p300 (EP300), or steroid receptor coactivator (SRC1), and histone acetyl transferases (HAT) to assemble a permissive complex on target gene promoters or enhancers, what results in focal chromatin relaxation and enhanced transcription of the cognate gene [2] (Figure 1). This is how PPARγ transactivates expression of a wealth of neuroprotective genes critical for mitochondria, microglial regulation and oxidative stress management [2]. Transrepression occurs differently depending on whether PPARγ is bound to a ligand or not. When PPARγ is unbound or bound to an antagonist (or a so-called inverse agonist), the PPARγ-RXRα dimer recruits corepressors as nuclear receptor corepressor 1 alpha (NCoR1α) or silencing mediator of retinoid and thyroid receptors (SMRT), and histone deacetylase 3 (HDAC3) to assemble a repressive complex on the target gene promoters, what impairs chromatin relaxation and inhibits transcription of the cognate gene [2]. PPARγ also exerts DNA-binding independent transrepression. When activated by an agonist, PPARγ bound to corepressors can bind to other transcription factors such as nuclear factor ĸB (NF-ĸB) or activating protein 1 (AP-1) to prevent them from activating inflammatory gene transcription [2] (Figure 1). The PPARγ-corepressor complex can also promote NF-ĸB degradation or export out of the nucleus [6,18]. These transrepressive mecanisms underlie the anti-inflammatory action of PPARγ [2].

Post-translational modifications regulate PPARγ activity. Ligand-bound PPARγ may undergo sumoylation which favours its stable binding to the corepressor [19]. PPARγ serine residues may be phosphorylated by the extracellular regulated kinases (ERK) or p38 MAP kinase pathways, what inhibits PPARγ activity by blocking ligand or cofactor binding [20]. In addition, PPARγ has been shown to undergo ubiquitination which increases its stability, or lysine acetylation which stabilizes its binding to co -activators or -repressors, or glycosylation with β-*O*-linked *N*-acetylglucosamine (*O*-GlcNAcylation) which decreases its transactivating ability [20].

PPARγ agonists and antagonists have been widely used in studies which shed light on PPARγ role in health and disease. The best known endogenous agonists of PPARγ are fatty acids such as 15-deoxy-∆^12,14^ prostaglandin (PG) J2 (15d-PGJ_2_), 15-hydroxyeicosatetraenoic acid (15-HETE), 9- or 13- hydroxyoctadecadienoic acid (9/13-HODE), all derived from oxidation cascades of poly-unsaturated fatty acids (PUFA) as linoleic acid or arachidonic acid [21,22]. Other natural PPARγ agonists are the phospholipids lysophosphatidic acid and hexadecylazelaoyl phosphatidylcholine, nitroalkenes and some dietary lipids such as isoflavones and flavonoids [23]. Recent studies have disclosed that astragaladoside IV from herbal extract [24], alliin from garlic [25] or cannabidiol from cannabis [26] were PPARγ agonists. Synthetic PPARγ agonists are thiazolidinediones (TZDs, e.g., rosiglitazone, pioglitazone, troglitazone) which share as common structural motifs a cyclic tail, an aromatic core, and an acidic head [23]. Noteworthy, the ability of TZDs to cross the brain blood barrier is controversial [6] and some receptor-independent effects of TZDs treatment have been reported [6,22]. Saroglitazar [27] and lanifibranor [28] were recently designed as efficient PPARγ agonists but they also activate PPARα and/or PPARβ/δ. In addition to activating the receptor, some ligands have been shown to upregulate PPARγ expression levels, like pioglitazone in embryonic rat brain cells [22], 15d-PGJ2 in rat primary microglia cells [29] and in a model of neonatal rat cerebral hemorrhage [30], and 9-HODE in human neural stem cells [31], in U937 monocytic cell line [32] and in kidney mesangial cells [33]. Structural studies have recently revealed that PPARγ so-called antagonists such as T0070907 [34] or the novel, rosiglitazone-derived, compound 3l [35] function as inverse agonists: their binding to PPARγ LBD results in conformational changes which increase the receptor affinity to corepressors and decrease its affinity to coactivators, what finally enhances PPARγ transrepressive activity.

## 3. PPARγ Expression in the Brain

In a founder study, in situ hybridization analyses of embryonic rat brains revealed transient PPARγ mRNA expression in forebrain, midbrain and, at higher levels, hindbrain, from E13.5, to before E18.5 [3]. Immunohistological exploration of PPARγ localization in the brain of adult rats revealed a heterogeneous pattern. PPARγ was detected in basal ganglia including substantia nigra, in hippocampus, in hypothalamus and in some parts of the cerebellum and of the cerebral cortex (cortex) [36]. Strikingly, in the latter, PPARγ expression appeared restricted to three out of the six cortical layers, and only in the frontal and parietal parts, suggesting a complex regulation of expression.

In the adult mouse brain, PPARγ immunoreactivity was observed specifically in prefrontal cortex, nucleus accumbens, amygdala and ventral tegmental area, four brain regions known to be involved in the pathophysiology of neurodegenerative diseases or of addiction [37]. In another study based on quantitative RTPCR and in situ hybridization on laser-microdissected mouse brain sections, PPARγ transcripts were detected in cortex, olfactory bulb and cerebellum, but not in caudate putamen or brain stem [38]. At the cell level, PPARγ was detected in both neurons and astrocytes of mouse or rat [36,37], and was only detectable in microglia after lipopolysaccharide (LPS) stimulation [37].

Few data are available on PPARγ expression in human brain due to its limited accessibility. We explored PPARγ expression by immunohistological analysis using fetal brain slices from elective abortion [31]. The cases were 23 to 28 gestational weeks and presented with conditions non related to brain such as (1) Digeorges syndrome (ie cardiopathy, endocrinopathy, facial dysplasia), (2) chorioamniotitis and anamnios (i.e., loss of amniotic liquid due to inflammation and premature rupture of membranes), (3) renal failure and (4) atrioventricular canal (heart dysplasia) and omphalocele (defective development of the abdominal wall). In any cases, no PPARγ was detected in any area of the brain parenchyma whereas it was detected in brain blood vessel cells. Soon after, immunofluorescence analysis of superior frontal gyrus (a part of the frontal cortex) from postmortem adult human brain has shown PPARγ expression in neurons and astrocytes but not in microglia [37]. Together those studies underscore that PPARγ is not evenly expressed in the brain, nor is it expressed in the same way in the fetal or adult brain, which raises the possibility that it exerts specific functions apart from its anti-inflammatory and metabolic functions.

## 4. PPARγ Responds to Specific Issues of the Brain Cell

### 4.1. Energy Supply, Oxidative Stress, and Mitochondria

The brain is particularly sensitive to changes in the energy supply: at baseline, the brain consumes over 20% of the oxygen and 25% of the glucose in the body, although it makes up only 2% of the body’s weight. This energy is dedicated to housekeeping neural cell functions, synaptic plasticity, neurotransmitter release and recycling, management of action and resting potentials, and neuronal computation and information processing [39]. Such high activity of energy metabolism and corresponding redox reactions lead to a high production of harmful reactive oxygen species (ROS) such as hydroxyl (HO^•^) and superoxide (^•^O_2_^−^) radical anions, hydroperoxyl radical (HO_2_^•^) and peroxyl radicals (ROO^•^) [40]. Neurons, as long lasting, postmitotic, cells, are more sensitive to the accumulation of oxidative damage in the long run as compared to dividing cells [41]. Thus, brain is highly sensitive to oxidative stress and this is exacerbated in neurodegenerative [40] or presumably psychiatric [39] disorders. PPARγ and/or PPARγ agonists were shown to exert antioxidant functions by upregulating the antioxidant enzymes haem oxygenase-1 (HO-1), catalase or copper/zinc superoxide dismutase (SOD) and downregulating the pro-oxydative enzymes inducible nitric oxide synthase (iNOS) or cyclooxygenase 2 (COX2) (reviewed in [5,7]). Rosiglitazone was also shown to prevent apoptosis related to amyloid [42] or tumor necrosis factor alpha (TNF-α) [43] in human neural stem cells by normalization of oxidative stress and mitochondrial function. Indeed, PPARγ protective role is further supported by its positive effect on mitochondria, that, beyond the cell powerhouse, are key regulators of redox balance [44]. A wealth of in vitro studies reviewed in [5,7] have shown that PPARγ and/or its agonists improved mitochondrial functions in human lymphocytes, adipocytes, astrocytes, neuroblastoma (SH-SY5Y) or neuronal (NT2) cell lines and hippocampal neurons, as shown by increased mitochondrial membrane potential (ΔΨ_m_), increased mitochondrial DNA (mtDNA) copy number, modulation of mitochondrial fusion-fission events and/or expression of factors beneficial to mitochondrial biogenesis and homeostasis, namely the co-activator PGC1-α [45], the mitochondrial transcription factor A (TFAM) [46] or the nuclear factor erythroid-derived 2-like 2 Nrf2 [47].

Recent studies have provided further insight on the role of PPARγ in an oxidative context in brain cells. Pioglitazone has been shown to inhibit, significantly for all, albeit moderately for some, the decrease in total thiol, SOD and catalase levels and the increase in malondialdehyde (MDA, a marker of PUFA peroxidation) levels in hippocampal and cortical extracts, in a rat model of hypothyroidism, a phenotype known to cause neurological damage [48]. Pioglitazone has also been shown to induce expression of TFAM and PGC-1α along with increased mitochondrial biogenesis and to restore mitochondrial membrane potential after challenge with rotenone, an inhibitor of the mitochondrial transport chain complex 1, in rat oligodendrocyte cultures [49]. Recent reports also documented a similar role of PPARγ and agonists in non brain tissues or cells. A C-terminally truncated form of PPARγ2 has been recently shown to localize in the mitochondrial matrix and to bind the D-loop region of mtDNA in brown adipocytes, what strongly suggested that PPARγ transactivated mitochondrial electron transport chain genes [50]. Lentivirally- expressed PPARγ has been shown to restore expression of the antioxidant uncoupling protein 1 (UCP1) in mouse tubular epithelial cells treated with hypoxia, concommitantly to inhibition of ROS generation, whereas pioglitazone administrated to mouse with experimental kidney hypoxia caused reduction of MDA levels and increase of UCP1 mRNA levels in kidney [51]. Pioglitazone has been also shown to increase catalase activity and levels of reduced gluthatione in a PPARγ-dependent manner in a rat model of hypertension [52]. Rosiglitazone was also found to decrease oxidative stress in MDCK canine kidney cells challenged with oxalate in a PPARγ-dependent way [53] and mitochondrial ROS levels, mitochondrial dysfunction and expression of the NLR family pyrin domain containing 3 (NLRP3) inflammasome in C_2_C_12_ myotubes and in a mouse model [54].

### 4.2. Neuroinflammation

Neuroinflammation represents the innate immune response specific to the nervous system. It is mediated by glial cells (i.e., astrocytes and the macrophage-like microglia cells), which activation underlies pathogenesis of neuroinflammation [55]. Noteworthingly, neuroinflammation is linked to oxidative stress since ROS are signaling messengers for inflammation [56]. Neuroinflammation has been widely documented and PPARγ and/or agonists have been shown to decrease neuroinflammation in a wealth of studies, as reviewed in [5].

Recent findings have provided better knowledge on the protective role of PPARγ in neuroinflammation. PPARγ has been shown to mediate suppression of inflammation by the anesthetic propofol in rat astrocytes [57]. To note, this effect is associated with PPARγ-dependent inhibition of the Wnt/β-catenin pathway, an important pathway which enhances neuroinflammation and has a mutual positive regulation with NF-ĸB [58]. It has been shown that translocator protein (TSPO) inhibited microglia activation by interleukin (IL-) 4 through PPARγ activity in a primary microglia polarization model [59]. Rice bran extract (which is rich in PUFA) as well as pioglitazone have been reported to protect against inflammation induced by lipopolysaccharides (LPS) in a mouse model, decreasing TNF-a and COX2 levels in brain, reducing striatal plaque formation and suppressing cortical and hippocampal tissue damage, all effects requiring PPARγ activity [60]. Other recent studies converged to support positive, PPARγ-dependent, role against neuroinflammation of PPARγ agonists as rosiglitazone which induced IL-10 in primary rat astrocytes exposed to LPS [61], or pioglitazone in a rat model of chronic intermittent hypoxia [62]. Other studies did not assess PPARγ involvement but still reported a protective role of its agonists against neuroinflammation, as rosiglitazone in a mouse model of epilepsy [63] and pioglitazone in rat models of autism [64], Parkinson’s disease [65], or neuroinflammation due to intracerebroventricular administration of LPS [66].

### 4.3. Neurogenesis

Brain is the most complex organ of the body, with a sophisticated tissue architecture. Neurogenesis and brain development rely on finely spatially and temporally tuned cell processes as differentiation, maturation, migration and acquisition of regional identities, whether they involve neural stem cells (NSCs), neural intermediate progenitor cells (NPCs) and/or their neuronal or glial progeny [67]. In the embryo, PPARγ has been shown to support NPC proliferation, trigger astrogliogenesis, inhibit neuron production (neuronogenesis) and enhance neurite outgrowth of differentiating neurons, whereas in the adult brain, PPARγ has been reported to enhance NSC self-renewal and differentiation [68]. A wealth of studies recently reviewed in [69] showed that PPARγ supports NSC growth, survival and stemness maintenance and positively regulates neuronogenesis and neurite outgrowth in maturing neurons. More recently, pioglitazone was shown to promote differentiation of rat primary oligodendrocytes [49].

Besides, neural progenitor/stem cells have specific metabolic needs: they have been shown to require predominantly glycolytic activity to maintain stemness and fatty acids as their energy source, whereas inhibition of lipogenic pathway was reported to decrease their proliferative potential [70]. Indeed, mitochondria are especially important in the regulation of NSC fate decisions, in embryonic and adult brains, as reviewed in [71]. It has been demonstrated that enhanced mitochondrial fragmentation was associated with increased levels of ROS which, as signalling messengers, promote Nrf2-mediated transcriptional upregulation of genes that activate differentiation and prevent self-renewal of NSCs [72]. By the way, a number of mitochondrial diseases or conditions with mitochondrial dysfunction result in neurological outcomes from mild cognitive impairment to severe psychiatric conditions [71]. Although these studies do not investigate the possible link between PPARγ and these processes, it is highly likely that the latter is involved given its importance for mitochondria and metabolism.

## 5. PPARγ in the Infected Adult or Developing Brain

Brain parenchyma can be infected by a large and heterogeneous range of viruses such as human immunodeficiency virus 1 (HIV), herpesviruses as herpes simplex virus (HSV), varicella-zoster virus (VZV), human cytomegalovirus (HCMV) or herpes virus 6 (HHV6 [73]), Zika virus (ZIKV), japanese encephalititis virus (JEV), West Nile virus (WNV) [11], Borna-disease virus [74] or SARS-CoV-2 [75]. However, the impact of viral infection on PPARγ activity has been investigated for only a small minority of these pathogens. In this respect, most of our knowledge comes from studies on HIV, ZIKV and HCMV infections.

### 5.1. PPARγ, the Adult Brain and Human Immunodeficiency Virus 1

Human immunodeficiency virus 1 (HIV, genus: *Lentivirus*, family: *Retroviridae*) bears a positive-sense, single-stranded RNA genome spanning around 9700 nucleotides and consisting of 9 genes encoding 19 proteins. HIV is predominantly transmitted by sexual contact across mucosal surfaces, by maternal-infant exposure in the absence of prophylaxis, or by percutaneous inoculation [76]. HIV infection is the causative factor of Acquired Immuno-Deficiency Syndrome (AIDS) that remains a major health issue worldwide [77]. Highly active anti-retroviral therapy (HAART) dramatically decreased mortality and morbidity of infected people through efficient inhibition of both viral replication and opportunistic infections, without, however, eradicating the virus from its lifelong latent reservoirs. A major consequence of persistent HIV infection is the development of HIV-Associated Neurocognitive Disorders (HAND), which are estimated to impact 30–60% of infected people [78], including individuals on successful HAART with undetectable plasma viral load [79]. Subjects with HAND may present paucisymptomatic neurocognitive impairment, or neurocognitive disorder with deficits in concentration, attention and memory, or HIV-associated dementia in the severely affected [80].

Resident brain cells show discrepant sensitivity to infection. Glia cells (astrocytes and microglia), but not neurons, are sensitive to HIV infection. Notably, two recent studies showed that microglial cells are highly permissive to HIV, i.e., they strongly support productive infection and virus spread [81], and that they constitute a stable population of slowly dividing, long-living (up to two decades) cells [82]. Together with other works reviewed in [83], those studies strongly suggested that microglia are the main HIV cell reservoir in the brain. In contrast, astrocytes were shown recently to be non permissive to HIV [79]. Upon infection, glial cells have been shown to release inflammatory cytokines (e.g., TNFα, interleukin-1β or interferon-γ), neurotoxic mediators (e.g., ROS, nitric oxide or glutamate) and viral proteins (namely « virotoxins », as the HIV glycoprotein gp120), resulting in an inflammatory, neurotoxic, and oxidative context, harmful and possibly lethal for neurons and deleterious for synaptic plasticity and astrocyte neuroprotective functions [84]. Unsurprisingly in this context, the anti-inflammatory action of PPARγ is found at the forefront and PPARγ agonists have been shown in a bundle of studies (reviewed in [22]) to be efficient regulators of microglia activation by inhibiting the synthesis of nitric oxide, prostaglandins, inflammatory cytokines and chemokines by microglia and by inducing apoptosis of activated microglia.

More recent studies have converged to highlight the beneficial role of PPARγ activation in HIV-infected brain. It has been disclosed that insulin treatment upregulated PPARγ expression in HIV-infected primary cultures of human microglia as well as in the cortex, but not in the striatum, of cats infected with feline immunodefiency virus, along with antiviral, anti-inflammatory, and neuroprotective outcomes [85]. Rosiglitazone was found to inhibit NF-κB as well as the release of inflammatory mediators (TNFα, IL-1β) or of iNOS and to prevent downregulation of the mouse ortholog of the glutamate transporter EAAT2 (excitatory amino acid transporter 2) caused by recombinant gp120 in primary mixed cultures of rat astrocytes and microglia or in rat after intracranial injection [86]. Interestingly, the same study reported a decrease in PPARγ transcript levels associated with gp120 treatment. EcoHIV is a chimeric HIV harboring gp80 from murine leukemia virus in place of gp120, thereby allowing for the infection of mouse cells and the onset of some molecular change observed in HAND [87]. Rosiglitazone and pioglitazone were demonstrated to reverse the increase in inflammatory mediators (TNFα, IL-1β, the chemokines CCL2, CCL3, CXCL10) and iNOS levels induced by EcoHIV in primary cultures of mouse glial cells and in mouse brains after intracranial injection [88]. In the same study, the two thiazolidinediones were also found to reduce in vivo EcoHIV p24 protein levels in the brain, what strongly supported an antiviral activity of the two agonists. Since then, similar results were obtained by the same group with the novel, non-thiazolidinedione, PPARγ agonist, INT131 [89]. PPARγ activity was however not assessed in these three reports.

Another role of PPARγ apart from neuroinflammatory modulation, has been highlighted in the context of HIV infection. Blood-brain barrier (BBB) is critical for HIV entry into the brain, and tight junction proteins are key structural and functional elements of integrity and efficiency of the BBB. In an in vitro BBB model, loss of barrier efficiency caused by HIV-infected human monocytes was shown to be reduced by overexpression of PPARγ in monocytes, in particular through repression of HIV-induced matrix metalloproteases (MMP) -2 and -9 activities [90]. Further, rosiglitazone has been shown to reduce astrogliosis, neuronal loss and disruption of BBB permeability caused by exposure to the HIV protein Tat, in a PPARγ-dependent fashion, in a mouse model [91]. Similarly, more recent works demonstrated that metabolites of the flavonoid quercetin suppressed MMP-2 activity and invasion of a lung cancer cell line in a PPARγ-dependent manner [92], and that PPARγ blocked the increase in activities of MMP-2 and MMP-9 due to *Toxoplasma Gondii* infection in astrocytes [93]. PPARγ could possibly hinder MMP expression by NF-ĸB transrepression since NF-κB has been shown to upregulate MMP-2 in murine melanoma cells [94] and MMP-9 in a rat model of intracerebral hemorrhage [95]. Those studies underscored the role of PPARγ in the management of both extracellular matrix and cell to cell adhesion.

On the virus side, NF-ĸB activity is known to be subverted to stimulate viral replication in the host cell by using the two NF-ĸB responsive elements within the promoter enhancer region of the long terminal repeat sequence (LTR) of the HIV genome [13]. Hence, by counteracting NF-kB through transrepression, PPARγ hampers not only inflammatory mediators release but also viral replication. Indeed, PPARγ activity was shown to suppress HIV LTR promoter activity, to decrease NF-κB occupancy of the LTR in infected cell, and, finally, to impair HIV replication in brain macrophages of an humanized mouse model of HIV encephalititis [96].

Together those studies converged to show that PPARγ has a beneficial role in the brain of HIV carriers, by counteracting both neuroinflammation and virus replication and by managing proteolysis-mediated regulation of the BBB.

### 5.2. PPARγ, the Developing Brain and Zika Virus

Zika virus (ZIKV, genus: *Flavivirus*, family: *Flaviviridae*) has a single-stranded RNA genome spanning around 11,000 nucleotides and consisting of a single open reading frame (ORF) and 5′ and 3′ noncoding regions. ZIKV is an arthropod-borne virus (*arbovirus*), predominantly transmitted by mosquitoes, but it can also be transmitted sexually or from mother to fetus [97]. Infected adults may present with mild symptoms or more severe neurological manifestations (eg Guillain-Barré Syndrome or encephalitis) whereas congenital infections may result in severe neurodevelopmental sequelae as microcephaly [97]. Although Zika pandemics outbreak in Brazil in 2016 is relatively recent, key findings on ZIKV neuropathogenesis have been published since. A wealth of recent studies have highlighted various neuropathogenic mechanisms of ZIKV infection, including neural cell receptors, altered gene expression, host RNA modifications or autophagy (reviewed in [97]). Brain organoid studies showed that ZIKV infection caused depletion of NPCs, because of either proliferation arrest and cell death or of premature differentiation (reviewed in [98]).

Notably, PPARγ transcript levels were found to be increased in human NPCs derived from induced pluripotent stem cells (iPSC), as revealed by RNA-seq, along with productive infection, proliferation arrest and apoptosis ([99], and supplemental data therein). A more recent study used quantitative proteomics and transcriptomics in ZIKV-infected human NPCs and revealed, however, decreased levels of PPARγ mRNA [100]. The same study reported upregulation of RXRγ, of a positive regulator of PPARγ activity (Signal transducer and activator of transcription [STAT] 5 [101]) and of two negative regulators of PPARγ activity (FGR, a member of the Src family of tyrosine protein kinases [102], and the AP-1 transcription factor c-Jun [103]), whereas nuclear receptor coactivator 1 (NCOA1), a coactivator of both RXR and PPARγ [104], was found to be downregulated.

Together the diversity of these regulations and their apparently contradictory consequences on PPARγ activity underscore the wide spectrum of cell signaling alterations caused by the infection. Those studies have paved the way to further investigations about the role of PPARγ in ZIKV infection of NPCs.

### 5.3. PPARγ, the Developing Brain and Human Cytomegalovirus

Human cytomegalovirus (HCMV, genus: *Cytomegalovirus*, family: *Herpesviridae*) is a beta herpes virus bearing a large genome (235-kb double stranded DNA) and that has remarkably co-evolved with humans. As all herpes viruses, it is able to establish lifelong latency after primo infection. Prevalence of HCMV ranges from 50–90% worldwide. HCMV is transmitted by body fluids. Although infection of immunocompetent adult subjects by HCMV is usually benign, congenital infection by HCMV is a leading cause of permanent abnormalities of the central nervous system [105]. About 1% of newborns are congenitally infected by HCMV each year in the U.S.A., as a result of either primary infection of a seronegative pregnant mother, or reinfection or viral reactivation in a seropositive pregnant mother. Among congenitally infected newborns, 10% are symptomatic at birth and present with neurological sequelae; in addition, 10 to 15% of those asymptomatic at birth will display neurological sequelae with onset later in infancy [106]. The most severely affected cases present with brain developmental abnormalities such as microcephaly or brain gyration defects whereas the most frequent sequelae include mental disabilities, sensorineural hearing or vision loss, and/or spastic cerebral palsies [105,106].

**Figure 2:**
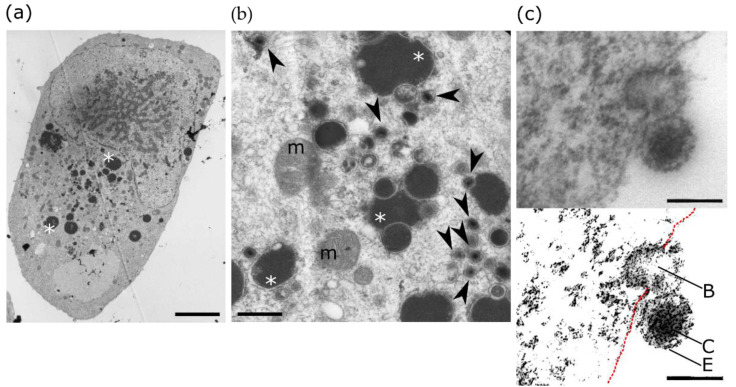
Transmission electron microscopy of human NSC cultures infected by HCMV. (**a**) Representative HCMV-infected NSC, containing numerous electron-dense lipid droplets (asterisks) consistent with active PPARγ. Scale bar: 5 µm. (**b**) Representative view of the cytoplasm of an infected NSC, containing morphologically mature viral particles (arrowheads) mitochondria (m) and still lipid droplets. Scale bar: 0.5 µm. (**c**) View of a HCMV particle shedding from an infected cell (top) and the same view after image processing (bottom) to highlight plasma membrane (red dotted line), the exocytosis cavity (B), the viral capsid (C) containing the electron-dense viral chromatin and the viral envelope (E). Scale bar: 0.2 µm.

Infection of neural progenitor cells in the developing brain is thought to be a primary cause of the neurological sequelae due to HCMV congenital infection ([31] and references therein). In vitro studies showed that HCMV infection of progenitors disrupted self-renewal and polarization [107], apoptosis [108], differentiation [107,108,109,110,111,112] or migratory abilities [113]. Because PPARγ had been shown previously to be upregulated in human placenta cells infected by HCMV [114], NSCs from human embryonic stem cells were used as a model to investigate the outcomes on PPARγ activity of the infection of neural progenitors by HCMV (Figure 2) [31].

Infection by HCMV was found to dramatically impair neuronal differentiation of NSCs [31]. PPARγ was barely detectable in uninfected NSCs whereas nuclei of infected NSCs showed strong immunoreactivity to PPARγ, indicating increased expression and activity of PPARγ [31]. This result was confirmed by chromatin immunoprecipitation, reporter gene assay or cellular lipid droplet staining. More importantly, this finding was strongly supported by the immunodetection of nuclear PPARγ specifically in the brain germinative zones of congenitally infected fetuses (N = 20) but not in control samples [31]. Lipidomic analysis revealed that levels of 9-HODE were significantly and specifically increased in infected NSCs, indicating that 9-HODE was the agonist associated with PPARγ activation. 9-HODE was also found to dramatically increase PPARγ levels and activity in uninfected NSCs, recapitulating the effect of infection [31]. Furthermore, 9-HODE treatment and/or single-out expression of PPARγ were sufficient to impair neuronogenesis of uninfected NSCs, whereas treatment of HCMV-infected NSCs with the PPARγ antagonist T0070907 restored a normal rate of differentiation [31]. Together these findings revealed that PPARγ exerts a negative role on NSC differentiation to neurons, should they be infected by HCMV or not. This has been supported soon after in another study which demonstrated that conditionally forced expression of Pparγ in mouse neural progenitors resulted in severe microcephaly and brain malformation [115].

The high level of 9-HODE biosynthesis could result from an interesting feature of HCMV particles. Indeed, the production of 9-HODE results from the oxidation of linoleic acid by cellular lipoxygenase 15-LOX, and linoleic acid is released from membrane phospholipids by viral, onboarded, phospholipase A2 (oPLA2) during infection. oPLA2 is a cell-derived phospholipase A2, packaged in the tegument of the virion during its release from the cell, and subsequently injected in the new host cell during viral particle entry [116]. In other words, HCMV particles carry oPLA2 as a ready to use tool for efficient 9-HODE biosynthesis in the host cell. HCMV infection has also been shown to inhibit Wnt/β-catenin signaling in dermal fibroblasts and placental extravillous trophoblasts [117], and this could also account for increased PPARγ activity in HCMV-infected NSCs since Wnt/β-catenin inhibits PPARγ [58].

Increased viral replication was observed in HCMV-infected NSCs exposed to 9-HODE [31]. Indeed, it had been demonstrated in human placenta cells that PPARγ exerted a positive role on HCMV replication by transactivating HCMV major immediate early promoter (MIEP) through the use of two PPREs [118]. Furthermore, neural progenitors require predominantly fatty acids as their energy source [119], and productive infection requires a large energetic supply and enhanced biosynthesis of fatty acids in the host cell to allow efficient viral replication and envelope assembly [120]. Increased PPARγ activity could thus be beneficial to both virus replication and host cell survival, given its role on fatty acid metabolism and mitochondria. This seems of particular importance in infection by HCMV since HCMV, as the other beta herpes viruses, undergoes in his host a long replicative cycle which numbers in days, and which, to be completed, requires prolonged survival of the host cell in spite of the metabolic storm caused by the infection.

## 6. Conclusions

Investigations about the outcomes of viral infection in the brain shed new light on PPARγ in the developing and adult brain. Recent studies underscored that expression and/or activity of such a master regulator as PPARγ must be finely tuned in time and space, especially during brain development.

**Figure 3:**
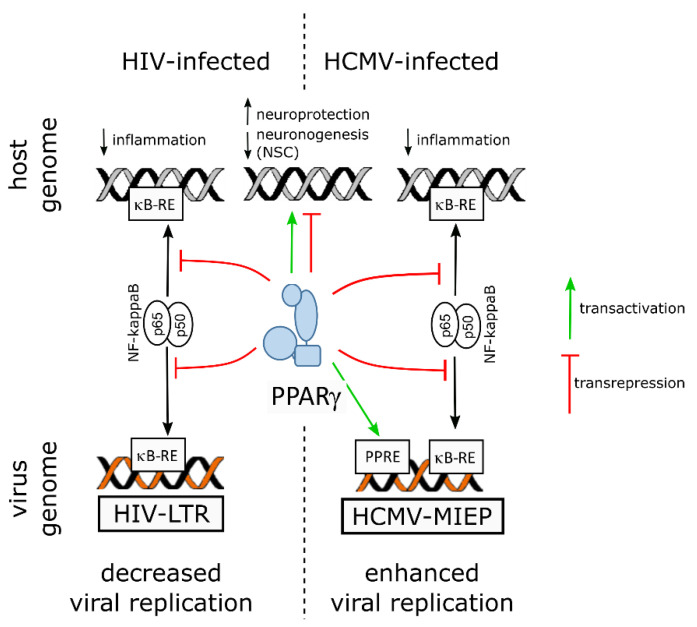
Graphical summary of PPARγ involvement during infection by HIV (**left**) or HCMV (**right**). In HIV-infected cells, PPARγ inhibits NF-kB by transrepression (red lines), thereby downregulating inflammatory genes and decreasing the efficiency of viral replication. In contrast, in HCMV-infected cells, PPARγ enhances viral replication by transactivation (green arrow) of the HCMV major immediate early promoter (MIEP) through two PPAR responsive elements (PPRE). In both cases, PPARγ regulates expression of the host cell genome, contributing to neuroprotection and, in neural stem cells (NSC), inhibition of neuronogenesis. ĸB-RE: NF-γB responsive element.

Probably because of its multifaceted role at the crossroads of inflammation, metabolism and cell differentiation, PPARγ can be a double-edged sword in viral infections of neural cells: besides its role in both moderating inflammation and supporting host cell survival, it can be deleterious to neuronal differentiation of progenitors, and either inhibit or support viral replication (Figure 3).

In the infected adult brain, the role of PPARγ in the host response to infection appeared beneficial against inflammation, oxidative stress and viral replication, as exemplified in HIV infection (Figure 3). PPARγ agonists have been proposed to be promising candidate drugs in the treatment of HIV-1 brain inflammation and neurocognitive outcomes [86], especially as they are already being used in treatment of HIV-associated lipodystrophy [121]. In contrast, in the developing brain, PPARγ activation has deleterious outcomes on neurogenesis, as shown in HCMV infection, and possibly in ZIKV infection. Notably, the activation of PPARγ in infection by HCMV is beneficial to viral replication (Figure 3).

Viruses undergo evolutionary pressure which optimizes both their spreading efficiency and the survival of their host. Whereas both the genomes of HIV and HCMV contain responsive elements to NF-ĸB, HCMV genome has evolved to gain two PPAR responsive elements within its major promoter. These responsive elements allow the subversion of PPARγ activity in the benefit of HCMV replication. Moreover, NF-ĸB transrepression by activated PPARγ accounts for immune evasion.

Yet, it is important to recall how variable the severity of neurological sequelae of HCMV infection may be. Host genetic factors still to be discovered may be important determinants of the severity of the sequelae, as, for example, cis-acting transcriptional regulators of PPARγ gene expression, or reciprocally, putative PPRE within PPARγ target genes.

## Data Availability

Not applicable.
